# Experimental data of biomaterial derived from *Malva sylvestris* and charcoal tablet powder for Hg^2+^ removal from aqueous solutions

**DOI:** 10.1016/j.dib.2016.05.032

**Published:** 2016-05-21

**Authors:** Alireza Rahbar, Sima Farjadfard, Mostafa Leili, Raheleh Kafaei, Vajiheh Haghshenas, Bahman Ramavandi

**Affiliations:** a Department of Nutrition, Faculty of Health, Bushehr University of Medical Sciences, Bushehr, Iran; b Department of Environmental Engineering, Graduate School of the Environment and Energy, Science and Research Branch, Islamic Azad University, Tehran, Iran; c Department of Environmental Health Engineering, School of Public Health, Hamadan University of Medical Sciences, Hamadan, Iran; d Environmental Health Engineering Department, Faculty of Health, Bushehr University of Medical Sciences, Bushehr, Iran; e Systems Environmental Health, Oil, Gas and Energy Research Center, Bushehr University of Medical Sciences, Bushehr, Iran

**Keywords:** Adsorption, Biomaterial, Hg^2+^ ion, *Malva sylvestris*, Charcoal tablet

## Abstract

In this experimental data article, a novel biomaterial was provided from *Malva sylvestris* and characterized its properties using various instrumental techniques. The operating parameters consisted of pH and adsorbent dose on Hg^2+^ adsorption from aqueous solution using *M. sylvestris* powder (MSP) were compared with charcoal tablet powder (CTP), a medicinal drug. The data acquired showed that *M. sylvestris* is a viable and very promising alternative adsorbent for Hg^2+^ removal from aqueous solutions. The experimental data suggest that the MSP is a potential adsorbent to use in medicine for treatment of poisoning with heavy metals; however, the application in animal models is a necessary step before the eventual application of MSP in situations involving humans.

**Specifications Table**

**Table:** 

Subject area	*Environmental Engineering*
More specific subject area	*Biomaterial*
Type of data	Image and figure
How data was acquired	– Mercury ions removal efficiency was determined based on mercury ion residue content in the filtered solution – pH meter (METLER TOLEDO FE20), Fourier Transform Infrared (FTIR) spectroscopy (Shimadzu 4300), atomic absorption spectroscopy (Atomic Absorption/Flame Emission Spectrophotometer Shimadzu AA-670).
Data format	Analyzed
Experimental factors	– *Malva sylvestris* powder (MSP): The MSP was prepared from leaves of *Malva sylvestris* at a temperature of 350 °C. – Charcoal tablet powder (CTP): The charcoal tablet was supplied from a pharmacy and grinded and powdered to obtain charcoal tablet powder (CTP). – Data of MSP and CTP were collected for Hg^2+^ adsorption from solution at identical conditions. – The data related to effects of adsorbents dose and solution pH was acquired.
Experimental features	Biomaterial from *Malva sylvestris* for Hg^2+^ removal
Data source location	*Bushehr University of Medical Sciences, Bushehr, Iran, GPS: 28.9667°N, 50.8333°E*
Data accessibility	*Data are available with the article*

**Value of the data**

• A simple method was used for providing a biomaterial form *Malva sylvestris* for adsorbing Hg^2+^ ion from aqueous solution.
• The data of *M. sylvestris* powder (MSP) and charcoal tablet powder (CTP) for Hg^2+^ removal from aqueous solution was described.
• This data set will be of value to the scientific community wanting to analyze the ability of MSP for treating the poisoning with heavy metal in animal models.
• MSP will be useful for wide range of mercury contaminated waters and wastewaters as it has good performance in around neutral pH and most of the waters and wastewaters have a neutral pH.

## 1 Data

**Fig. 1:**
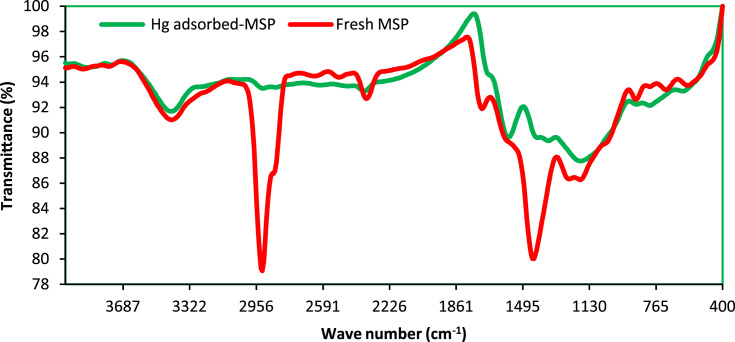
FTIR spectra of MSP at wave numbers from 400 to 4000 cm^−1^.

**Fig. 2:**
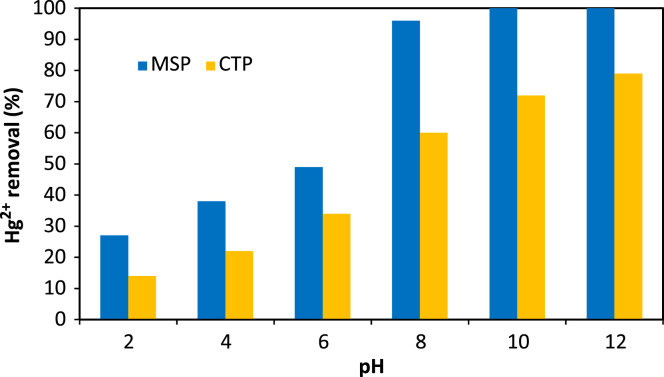
Effect of pH on Hg^2+^ adsorption by MSP and CTP (Hg^2+^ concentration: 5 mg/L; MSP and CTP dose: 1 mg/L; contact time: 40 min).

**Fig. 3:**
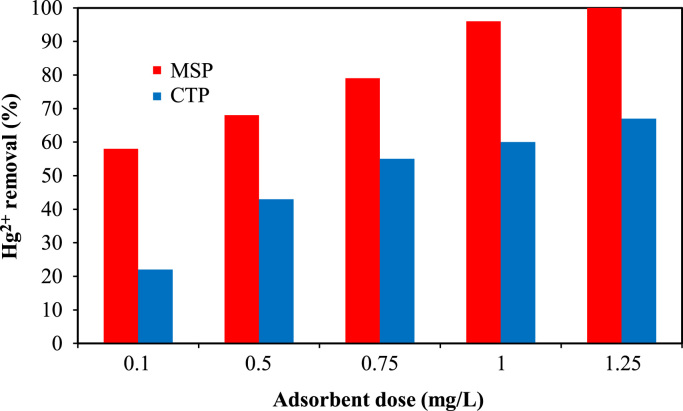
Effect of adsorbent dose on Hg^2+^ removal (Hg^2+^ concentration: 5 mg/L; solution pH: 8 mg/L; contact time: 40 min).

Data analysis indicated that MSP particles had a BET multipoint surface area of 2.34 m^2^/g and a total pore volume at 0.9925*P*/*P*_0_ of 0.0002 cm^3^/g. The FTIR of the fresh and Hg^2+^-loaded MSP particles at wave numbers from 400 to 4000 cm^−1^ are shown in Fig. 1. The data of the effects of solution pH on the adsorption percentage of mercury ions by CTP and MSP is presented in Fig. 2. The data acquired for adsorption of mercury ions by different doses of CTP and MSP is also depicted in Fig. 3.

## 2 Experimental design, materials and methods

### 2.1 Materials

The *Malva sylvestris* powder (MSP) was prepared from leaves of *M. sylvestris* as follows: First, *M. sylvestris*, was provided from the local area around of Bushehr city, Iran. Then the samples of *M. sylvestris* were air-dried for four consecutive days and powdered using a grinder to obtain the particle size of mesh 200 (=0.074 mm). After that, the prepared powder was put an oven at a temperature of 350 °C under air and kept at this temperature for 2 h. Finally, the resulting MSP was put in a capped-glass vessel for use as an adsorbent as required. The charcoal tablet was supplied from a local pharmacy. The tablet was grinded and sieved to mesh size of 200 to obtain charcoal tablet powder (CTP).

### 2.2 Experimental procedure

Hg^2+^ removal tests with the prepared MSP and CTP were conducted in 100-mL flask while agitating on a shaker–incubator instrument (Parsazma Co., Iran). Each test contained of preparing 50 mL of Hg^2+^ solution with a given initial concentration, and the initial pH of the solution was adjusted by adding 0.1 M HCl and NaOH solutions. Thirty milli-liters aliquots of the samples were then filtered through 0.45-µm filters at determined time intervals. The percentage of removal and the amount of adsorption at equilibrium, *q_e_* (mg/g), by MSP and CTP were obtained by the following equations [1], [2]:

(1) $$adsorption\;(\%)=\frac{C_{0}-C_{f}}{C_{0}}\times 100$$

(2) $$q_{e}=\frac{C_{0}-C_{e}}{W}\times V$$

where *C*_0_, *C_f_*, and *C_e_* (mg/L) are adsorbate concentrations at initial, final, and equilibrium, respectively. *V* (L) is the solution volume and *W* (g) is the dry mass of adsorbents.

All tests were done in triplicate to ensure the reproducibility of data, and the average values are stated. Control tests containing no MSP or CTP were also performed.

### 2.3 Adsorbent characterization and analytical methods

The specific surface area based on the Brunauer, Emmett, and Teller (BET) method. The pore volume was calculated by Micromeritics/Gemini-2372. The mean pore diameter was determined using BET, and the total pore volume was measured using the method reported by Altenor et al. [3]. The functional groups present on the surface of the MSP were identified by Fourier Transform Infrared (FTIR) spectroscopy (Shimadzu 4300). The concentration of Hg^2+^ ion in solutions was measured using atomic absorption spectroscopy (Atomic Absorption/Flame Emission Spectrophotometer Shimadzu AA-670). The solution pH was determined using a pH meter (METLER TOLEDO FE20). The paired *t*-test statistical analysis at a 95% confidence level (*p*<0.05) was used to compare the Hg^2+^ removal efficiency of MSP and CTP. The statistical analysis was performed using SPSS software version 22.
